# Structural modeling of a novel *SLC38A8* mutation that causes foveal hypoplasia

**DOI:** 10.1002/mgg3.266

**Published:** 2017-02-26

**Authors:** Marcus A. Toral, Gabriel Velez, Katherine Boudreault, Kellie A. Schaefer, Yu Xu, Norman Saffra, Alexander G. Bassuk, Stephen H. Tsang, Vinit B. Mahajan

**Affiliations:** ^1^ Omics LaboratoryUniversity of IowaIowa CityIowa; ^2^ Department of Ophthalmology and Visual SciencesUniversity of IowaIowa CityIowa; ^3^ Medical Scientist Training ProgramUniversity of IowaIowa CityIowa; ^4^ Department of OphthalmologyUniversity of MontrealMontrealQuebecCanada; ^5^ The Barbara & Donald Jonas Laboratory of Regenerative Medicine and Bernard & Shirlee Brown Glaucoma LaboratoryDepartments of Ophthalmology, Pathology & Cell BiologyCollege of Physicians & SurgeonsColumbia UniversityNew York CityNew York; ^6^ Edward S. Harkness Eye InstituteNew York‐Presbyterian HospitalNew York CityNew York; ^7^ Department of OphthalmologyMaimonides Medical CenterBrooklynNew York; ^8^ Department of PediatricsUniversity of IowaIowa CityIowa

**Keywords:** foveal hypoplasia, OCT‐angiography, precision medicine, SLC38A8, structural modeling

## Abstract

**Background:**

Foveal hypoplasia (FH) in the absence of albinism, aniridia, microphthalmia, or achromatopsia is exceedingly rare, and the molecular basis for the disorder remains unknown. FH is characterized by the absence of both the retinal foveal pit and avascular zone, but with preserved retinal architecture. *SLC38A8* encodes a sodium‐coupled neutral amino acid transporter with a preference for glutamate as a substrate. SLC38A8 has been linked to FH. Here, we describe a novel mutation to *SLC38A8* which causes FH, and report the novel use of OCT‐angiography to improve the precision of FH diagnosis. More so, we used computational modeling to explore possible functional effects of known SLC38A8 mutations.

**Methods:**

Fundus autofluorescence, SD‐OCT, and OCT‐angiography were used to make the clinical diagnosis. Whole‐exome sequencing led to the identification of a novel disease‐causing variant in *SLC38A8*. Computational modeling approaches were used to visualize known SLC38A8 mutations, as well as to predict mutation effects on transporter structure and function.

**Results:**

We identified a novel point mutation in *SLC38A8* that causes FH. A conclusive diagnosis was made using OCT‐angiography, which more clearly revealed retinal vasculature penetrating into the foveal region. Structural modeling of the channel showed the mutation was near previously published mutations, clustered on an extracellular loop. Our modeling also predicted that the mutation destabilizes the protein by altering the electrostatic potential within the channel pore.

**Conclusion:**

Our results demonstrate a novel use for OCT‐angiography in confirming FH, and also uncover genotype–phenotype correlations of FH‐linked *SLC38A8* mutations.

## Introduction

The recent success of regenerative medicine approaches for retinal disease makes accurate and precise diagnosis even more important in children with inherited disease, who may become candidates for gene and cell therapies (Zhou et al. 1989; Li et al. 2014, 2016; Tsang et al. 2014; Lin et al. 2015; Yang et al. 2015; Zheng et al. 2015; Pyo Park et al. 2013). Precision medicine requires highly accurate phenotype–genotype correlations that could be enhanced with new imaging and DNA sequencing modalities. For example, children with nystagmus and vision loss present a diagnostic challenge.

The solute carrier (SLC) family of channel proteins is a superfamily of transporter proteins that is comprised of 43 families and 298 genes. The SLC38 subfamily, in particular, are sodium‐coupled neutral amino acid transporters (SNATs) (Hagglund et al. 2015). The SLC38 isoforms are ubiquitously expressed, but SLC38A8 (SNAT8) is expressed predominantly in the central nervous system and retina, and has been associated with the FHONDA (OMIM #609218) syndrome, characterized by foveal hypoplasia, optic nerve misrouting, and anterior segment dysgenesis in the absence of pigmentation defects (Al‐Araimi et al. 2013; Poulter et al. 2013; Perez et al. 2014).

Here, we report the novel use of OCT‐angiography to diagnose foveal hypoplasia and exome sequencing to identify a novel variant in the *SLC38A8* gene (OMIM #615585). Although mutations in *SLC38A8* were previously described, their disease‐causing mechanisms are poorly understood. We used structural modeling to gain insight into the effects of the *SLC38A8* mutations and examine genotype‐proteotype correlations. We showed that our p.Asp283Ala substitution mutation occurs on an extracellular loop of SLC38A8, close to two other known mutations. Furthermore, we characterized the effects of all known SLC38A8 mutations on protein stability, predicting ours to act as a destabilizer. Finally, we showed that our mutation alters the predicted electrostatic potential within the channel pore, providing further insight into how our mutation may alter channel function.

## Methods

Ethical compliance: individuals from a single nonconsanguineous Ashkenazi Jewish family were evaluated for this study and data collection used in this study was approved by the Institutional Review Board for Human Subjects Research at Columbia University Medical Center (AAAB6560), was compliant with the Health Insurance Portability and Accountability Act, and adhered to the tenets of the Declaration of Helsinki. Written informed consent was received from participants. Clinical examination and genetic testing was performed as previously described (Bassuk et al. 2014; Duncker et al. 2014; Moshfegh et al. 2016). Fundus autofluorescence images and SD‐OCT were obtained using Spectralis Heidelberg (Heidelberg, Germany). OCT‐angiography images were obtained using the Zeiss Angioplex OCT‐A on the Cirrus HD‐OCT Model 5000 Version 9.50.8211 (Carl Zeiss Meditec, Jena, Germany) using Zeiss Optical Micro Angiography (OMAG^C^) Algorithms. Detailed methods for primary sequence analysis, whole‐exome sequencing, structural modeling and analysis are reported as online supporting information. *SLC38A8* GenBank accession and version number: NG_034136.1.

## Results

**Figure 1:**
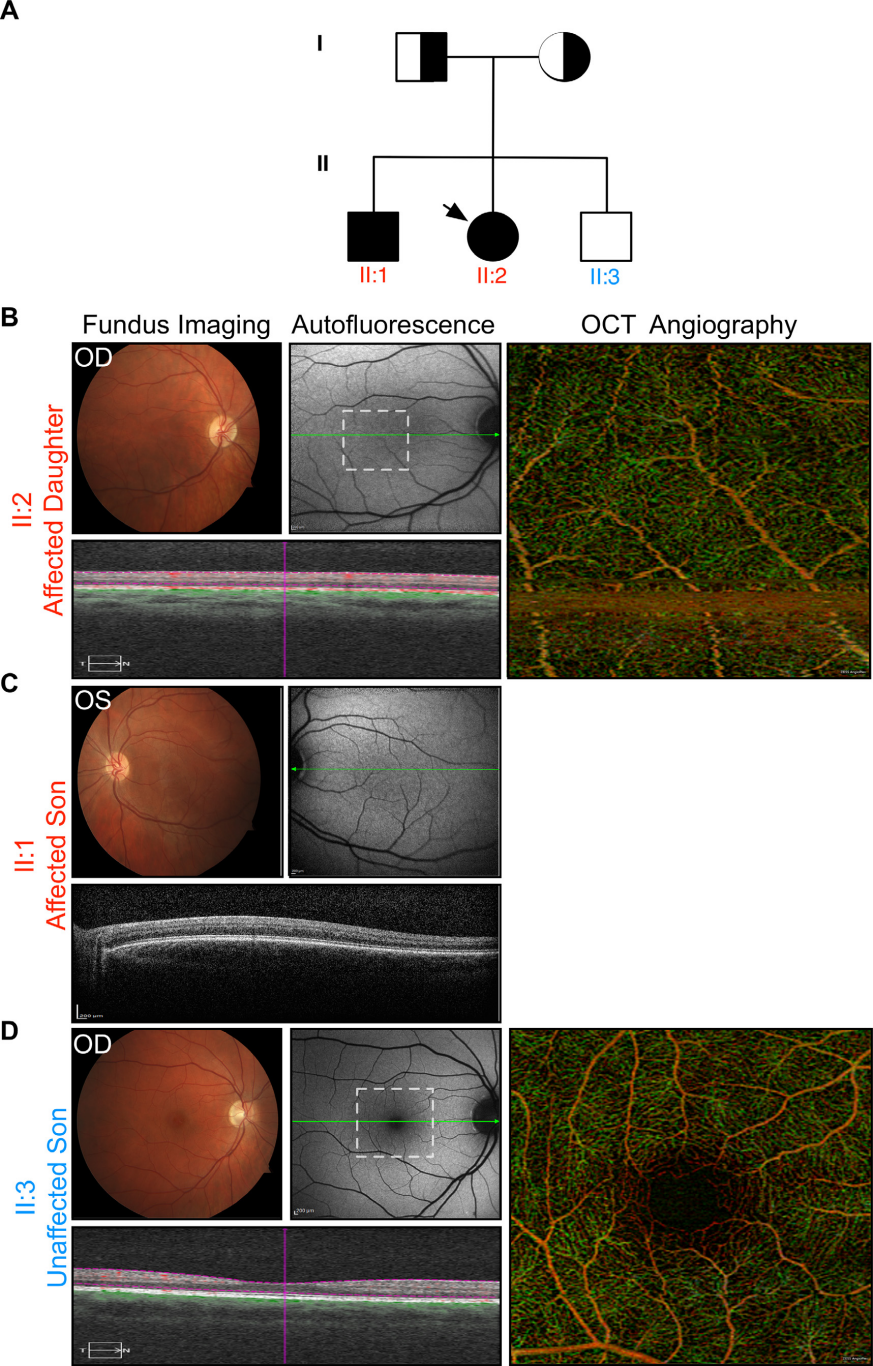
Clinical imaging. (A) Family pedigree shown. Exome sequencing revealed heterozygous mutation to *SLC38A8* (OMIM #615585) in the mother, father, and homozygous mutations in the affected daughter (proband; indicated with arrow) and son. The variant was not present in either allele for the unaffected son. For clinical images, findings were similar bilaterally and best images, for either right eye (OD) or left eye (OS), were chosen. (B) OD Fundus imaging, autofluorescence, OCT, and OCT‐angiography shown for proband case (II:2). Region of OCT‐angiography indicated by white dotted‐line box. Note, the absence of foveal pit, fundus autofluorescence revealing an abnormal intensity of lipofusin and pigment in the macular region, and absence of the foveal avascular zone. Normal retinal architecture preserved. (C) OS imaging of affected son (II:1) reveals signs of foveal hypoplasia similar to proband (sister). OCT‐angiography could not be obtained due to severity of nystagmus. (D) OD imaging of unaffected son (II:3) shows normal retinal and foveal architecture and morphology. Foveal pit, foveal avascular zone, and normal foveal autofluorescence present.

The proband (II:2) was a 32‐year old female with mild nystagmus and a best‐corrected Snellen visual acuity of 20/80‐2 OD and 20/80‐1 OS. Her 28‐year old brother (II:1) was also affected. He had nystagmus and his visual acuity measured 20/100 OD and 20/150 OS. Both the affected sister and brother had moderate hyperopia (+3 diopters) and astigmatism (+2 diopters, vertical meridian). Their developmental history and neurological examinations were normal. Their eldest brother (II:3) and parents (I:1, I:2) were unaffected with normal visual acuities and no nystagmus. The inheritance pattern suggested an autosomal recessive condition (Fig. 1A).

Fundus examinations of the affected siblings demonstrated a poorly defined foveal avascular zone and blunt foveal reflexes bilaterally. There was no intraretinal pigmentary migration, pisciform flecks, optic disk pallor, or retinal vessel attenuation. Fundus autofluorescence (AF) revealed an abnormal intensity of lipofusin and pigment in the macular region (Fig. 1B,C). Additionally, no iris transillumination was observed. Spectral domain optical coherence tomography (SD‐OCT) suggested the absence of a foveal pit, yet preserved overall retinal structure. To examine foveal morphology even more closely, OCT‐angiography was performed. Motion artifacts in the younger male sibling prevented adequate imaging. However, these studies in the elder sibling confirmed absence of the foveal avascular zone, showing retinal vessels penetrating into the normally avascular foveal region (Fig. 1B). Together, these studies unequivocally confirmed foveal hypoplasia.

To broadly interrogate the genome, we performed whole‐exome sequencing. The most common cause of foveal hypoplasia is albinism, but no mutations were identified in *SLC45A2, TYR, TYRP1, GPR143, OCA1, OCA2, OCA3, or OCA4*. However, we did find a homozygous coding mutation (c.848A>C, p.Asp283Ala) in the *SLC38A8* gene of both affected individuals. The unaffected sibling did not carry this variant, and both parents were heterozygous at this locus. There was only one homozygous p.Asp283Ala mutation observed in the ExAC dataset of over 60,000 individuals (who are not necessarily screened for eye disease). The patient variant was not reported in the 1000 Genomes database.

**Figure 2:**
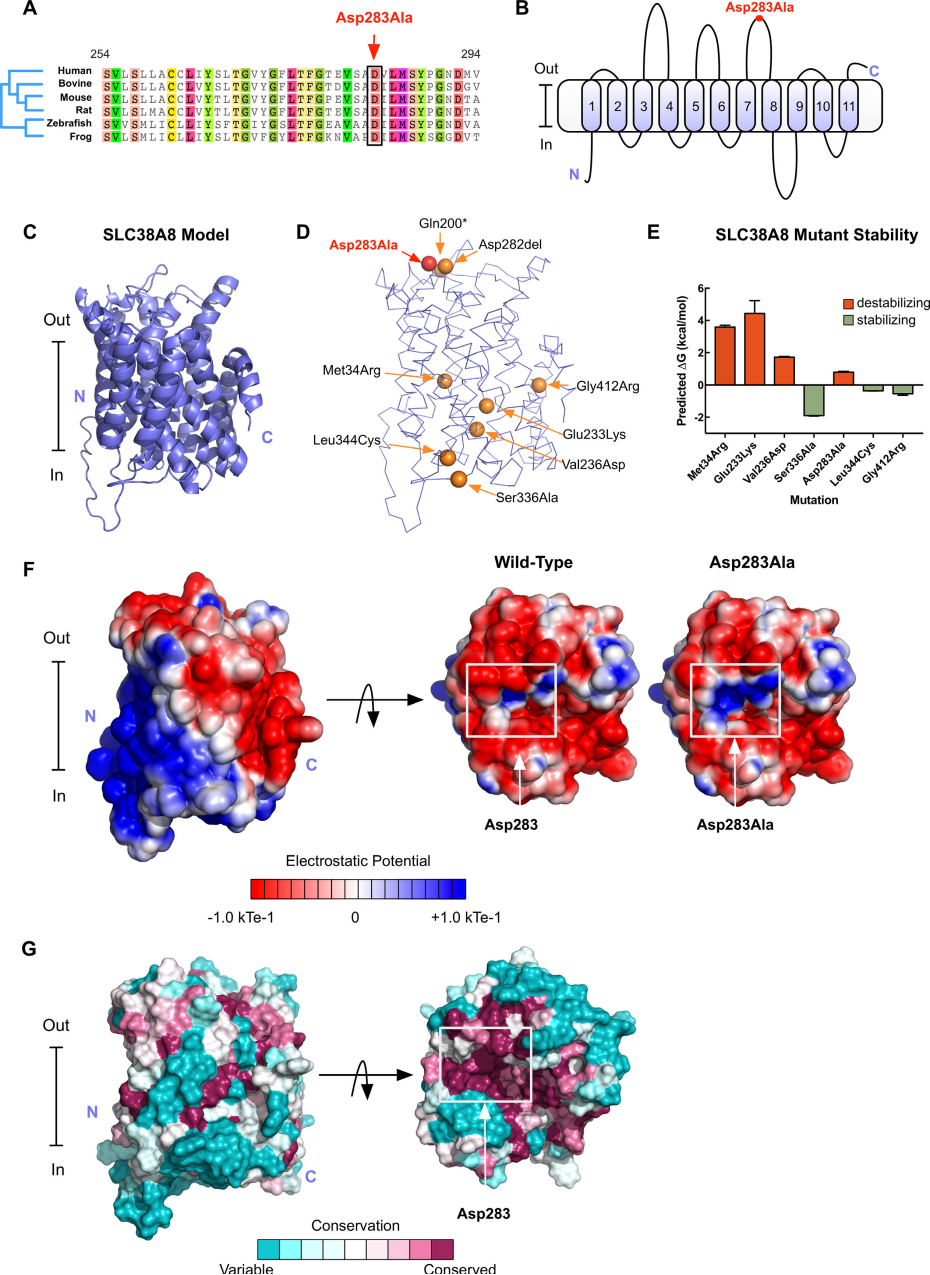
Structural modeling of patient SCL38A8 mutations: (A) Sequence alignment showing the p.Asp283 position to be conserved across multiple species. (B) Membrane topological structure diagram predicts SLC38A8 to have 11 transmembrane helices and places the p.Asp283Ala mutation on the extracellular surface of the channel. (C) Our SLC38A8 model generated using the I‐TASSER program. (D) Known FHONDA‐associated mutations mapped onto our SLC38A8 model. (E) Mutations were introduced into our model using FoldX and predicted changes in total energy were calculated. Positive changes in total energy were predicted to be destabilizing. (F) Electrostatic potential surface of our SLC38A8 model shows a more positive potential surface at the site of the mutation near the opening of the channel pore. (G) Consurf coloring of the SLC38A8 model reveals the p.Asp283 residue to be 100% conserved across 103 homologous sequences.

While this specific variant has not previously been associated with foveal hypoplasia, several mutations in *SLC38A8* have been reported to cause foveal hypoplasia (Poulter et al. 2013; Perez et al. 2014). *SLC38A8* encodes a sodium‐coupled neutral amino acid transporter (SNAT) protein, which predominantly functions as a glutamate transporter (Hagglund et al. 2015). A primary sequence analysis in PolyPhen‐2 (Adzhubei, et al., 2013), SIFT (Sim et al., 2012), and PROVEAN (Choi and Chan, 2015) predicted the p.Asp283Ala mutation to have deleterious effects on SLC38A8 function (Tables S1–S3) and a multiple sequence alignment revealed the p.Asp283 residue to be conserved across multiple species (Fig. 2A).

Although our primary sequence analysis predicted this mutation to be damaging, it did not give insight into its mechanism of pathogenicity. In the absence of a functional assay, we have used structural modeling to help determine the pathogenicity of gene mutations (Bassuk et al. 2015; Moshfegh et al. 2016), and did so for *SLC38A8* mutations. We started by modeling the transmembrane topological structure based on hydropathy, using TMHMM (Krogh et al., 2001). This analysis predicted SLC38A8 to have 11 helices and placed the p.Asp283 residue on the extracellular side of the membrane (Fig. 2B). We then modeled the SLC38A8 tertiary structure using a threading approach (Zhang, 2008; Roy et al., 2010; Yang et al. 2015; Yang and Zhang, 2015)). The generated model matched the membrane topological predictions made in TMHMM (Fig. 2C).

Using our structural model, we mapped the p.Asp283Ala mutation, as well as other known FHONDA mutations, onto the SLC38A8 structure (Fig. 2D) (Poulter et al. 2013). Mutational analysis in FoldX (Schymkowitz et al. 2005) predicted the p.Asp283Ala mutation to be mildly destabilizing compared to previously identified mutations, p.Met34Arg, p.Glu233Lys, and p.Val236Asp (Fig. 2E). Therefore, we looked at other features of the mutation to determine its pathogenicity. The p.Asp283Ala mutation localized to the extracellular side of the channel, in close proximity to two other known FHONDA mutations (p.Ala282del and p.Gln200*). This substitution from a polar, negatively ‐charged residue to a hydrophobic, uncharged residue may have effects on local sodium concentration at the extracellular side of the channel given the mutation's proximity to the channel pore. Therefore, we sought to model the predicted effects of the mutation on the channel's calculated electrostatic potential using the linearized Poisson–Boltzmann equation in APBS (Baker et al. 2001). As expected, the p.Asp283Ala mutation created a more positive electrostatic potential at the extracellular side of the channel, potentially disrupting the local concentration of sodium and affecting glutamine transport (Fig. 2F). Further analysis using ConSurf revealed the p.Asp283 residue to be highly conserved across 103 unique homologous sequences (100%), highlighting the importance of this residue in SLC38A8 function (Fig. 2G) (Ashkenazy et al. 2010).

We further used our SLC38A8 model to determine the pathogenicity of previously published FHONDA mutations and compare their predicted mechanisms of pathogenicity to p.Asp283Ala (Fig. S2). Two mutations (p.Gln200* and p.Ala282del) result in the deletion of one or multiple amino acids, thereby affecting localization of the SLC38A8 channel to the membrane (Fig. S2C and S2F). Interestingly, these two mutations are located in close proximity to p.Asp283Ala (Fig. 2D). Three mutations were predicted to affect stability of the folded protein by affecting hydrogen‐bonding (p.Glu233Lys; Fig. S2D), removing hydrophobic residues near the channel pore (p.Val236Asp; Fig. S2E), or altering the hydropathy near the protein‐membrane interface (p.Met34Arg; Fig. 2B). Three mutations were located on the intracellular side of the channel (p.Ser336Ala, p.Leu344Cys, and p.Gly412Arg; Fig. S2G–I). ConSurf analysis revealed the p.Gly412 residue to be conserved (95%) while p.Ser336 were p.Leu344 residues were variable among homologous SLC sequences (2% and 9%, respectively). The mechanisms of these three mutations are unknown, and our analysis in FoldX predicted them to be stabilizing (Fig. 2E), suggesting the need for an SLC38A8 functional assay to determine their pathogenic effect.

## Discussion

The fovea is the site of high‐resolution vision, and is characterized morphologically by a foveal avascular zone, increased density of cone photoreceptors, and excavation of inner retinal neurons (Dubis et al. 2012). During human foveal development, cones become tightly packed before elongating and migrating centripetally, leaving behind a shallow foveal pit in their wake (Hendrickson and Yuodelis 1984; Yuodelis and Hendrickson 1986; Diaz‐Araya and Provis 1992). In humans, this pit first forms by around 24–26 weeks postmenstrual age (Dubis et al. 2012), though the site of its future formation can be identified by morphological (Provis et al. 1998) and molecular characteristics (Cornish et al. 2005; Kozulin et al. 2010) as early as 12 weeks postmenstrual age (Dubis et al. 2012).

Foveal hypoplasia is defined by the absence of the foveal pit, where all retinal layers and retinal vasculature continue through the presumed foveal region (Perez et al. 2014). It is unclear whether foveal hypoplasia is a morphological defect of developmental mis‐organization. Clinically, detection of foveal hypoplasia can be very challenging. Here, we used OCT‐angiography to more clearly show retinal vessels penetrating through the presumed foveal avascular zone. The use of current OCT‐angiography imaging can be subject to motion artifacts in cases of nystagmus. To accommodate for nystagmus in OCT‐angiography imaging, we attempted to image subjects at their null point. However, in cases of severe nystagmus, it may not be possible to capture OCT‐angiography images of sufficient quality until acquisition speeds are improved. Nevertheless, combined with OCT and autofluorescence, we confirmed the finding of foveal hypoplasia. While a number of conditions can result in foveal hypoplasia, including aniridia and albinism, this finding in the absence of well‐known syndromes is rare. Resulting from mutations to *SLC38A8,* FHONDA syndrome (OMIM #609218) is a recently described, recessively inherited disease defined by foveal hypoplasia, optic nerve decussation defects, and anterior segment dysgenesis (Al‐Araimi et al. 2013; Perez et al. 2014; Hagglund et al. 2015). Notably, our novel mutation did not reveal all signs of FHONDA, as patients lacked signs of anterior segment dysgenesis (optic nerve misrouting was not evaluated). Interestingly, our structural modeling revealed our p.Asp283Ala mutation to occur on an extracellular loop on SLC38A8, closely adjacent to two other reported mutations known to produce FHONDA (Poulter et al. 2013). These two other mutations produce substantial deletions in the protein, while our mutation was just an amino acid substitution. We speculate that our less‐severe alteration may be why we observed isolated foveal hypoplasia without significant anterior segment abnormalities in our case, but more severe FHONDA syndrome was seen in the two deletions. Thus, of the signs of FHONDA, foveal hypoplasia may be the defect which is most sensitive to SLC channel abnormalities, where even minor alterations to channel conductivity can alter retinal development.

Finally, while our structural model provided useful insights in this study, it is important to note that the SLC38A8 channel has <30% sequence homology with known templates in the protein database (PDB). When sequence homology to available templates in the PDB is less than <30%, selection of a suitable template for structural modeling can be difficult. To overcome the absence of a complete structural template, threading and ab initio modeling methods like I‐TASSER can match fragments of the protein sequence onto the 3D structure of other solved proteins. The fragments are then used to assemble structural conformations that are then further refined by ab initio modeling of loop regions and molecular dynamics simulations. Using this approach, we produced a useful structural model for SLC38A8 that was consistent with homology‐based models and membrane topological predictions.

Here, we have described a novel mutation in *SLC38A8* which produces isolated foveal hypoplasia, without evidence of pigmentation defects or significant anterior segment dysgenesis. This case demonstrates OCT‐angiography as a powerful diagnostic tool for the identification and characterization of foveal hypoplasia. More so, through whole‐exome sequencing and structural modeling, we were able to describe the location of this new mutation and place it in context with other known channel mutations.

## Conflict of interest

The authors declare no conflict of interest.

## Supporting information


**Figure S1.** Structural models of SLC38A8.

**Figure S2.** Modeling of known FHONDA mutations.

**Table S1.** Prediction of the effects of SLC38A8 mutations using the PolyPhen‐2 server.
**Table S2.** Prediction of the effects of SLC38A8 mutations using the SIFT server.
**Table S3.** Prediction of the effects of SLC38A8 mutations using the PROVEAN server.
**Appendix S1.** Supplemental methods.
