# Supplementary figures and images for: Structural modeling of a novel SLC38A8 mutation that causes foveal hypoplasia
Source: Mol Genet Genomic Med. 2017 Feb 26;5(3):202–9. doi: 10.1002/mgg3.266 (PMC5441399; doi:10.1002/mgg3.266)

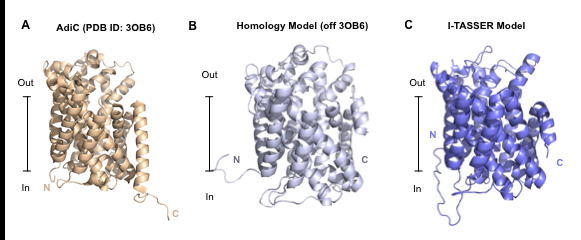

Supplement: Supplementary file 1 — Figure S1. Structural models of SLC38A8. [file MGG3-5-202-s001.tiff]

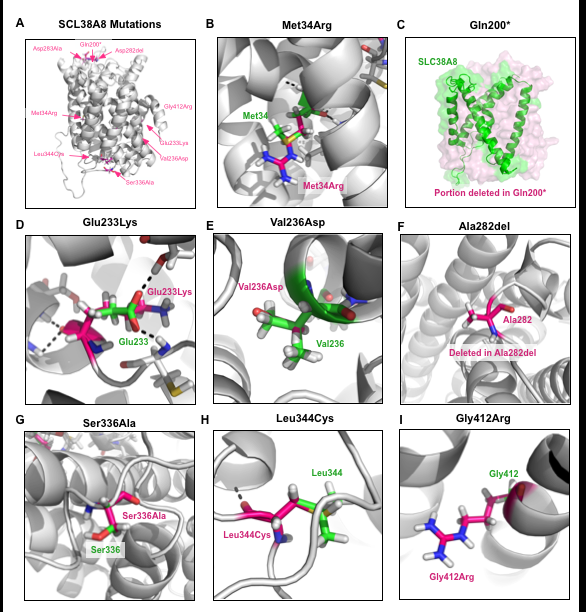

Supplement: Supplementary file 2 — Figure S2. Modeling of known FHONDA mutations. [file MGG3-5-202-s002.tiff]
