# Supplementary material for: Structural modeling of a novel SLC38A8 mutation that causes foveal hypoplasia
Source: Mol Genet Genomic Med. 2017 Feb 26;5(3):202–9. doi: 10.1002/mgg3.266 (PMC5441399; doi:10.1002/mgg3.266)
Supplement: Supplementary file 3 — Table S1. Prediction of the effects of SLC38A8 mutations using the PolyPhen‐2 server. Table S2. Prediction of the effects of SLC38A8 mutations using the SIFT server. Table S3. Prediction of the effects of SLC38A8 mutations using the PROVEAN server. Appendix S1. Supplemental methods. [file MGG3-5-202-s003.docx]

**Supplemental Data**

**Figure S1. Structural models of SLC38A8: (A)** Crystal structure of bacterial arginine/agmantine antiporter AdiC (PDB ID: 3OB6) was used a template to generate the homology-based model of SLC38A8 **(B)** Homology-based model of SLC38A8 based off AdiC (PDB ID: 3OB6). **(C)** I-TASSER-generated model of SLC38A8. This model was chosen for further analysis.

**Figure S2. Modeling of known FHONDA mutations: (A)** Location of FHONDA mutations on our SLC38A8 model. Mutated residues are highlighted in magenta and wild-type residues in green. **(B)** Location of p.Met34Arg mutation. This substitution from a hydrophobic to a positively-charged polar amino acid near the membrane may disrupt the association with membrane lipids. **(C)** Predicted effect of p.Gln200* mutation: this nonsense mutation is predicted to produce a truncated protein missing several transmembrane helices, thereby disrupting membrane localization. **(D)** Location and predicted effect of p.Glu233Lys mutation: p.Glu233 is predicted to form hydrogen bonds with several surrounding residues (p.Cys231 and p.Ser380), which are potentially disrupted in the mutant. **(E)** Location of p.Val236Asp mutation near the channel pore. This substitution from a hydrophobic to a negatively-charged polar amino acid near the center of the protein may affect its folding and stability. **(F)** Location of deleted residue in p.Ala282del mutant. **(G)** Location of p.Ser336Ala mutation. **(H)** Location of p.Leu344Cys mutation. **(I)** Location of p.Gly412Arg mutation. This residue is 94% conserved across 103 homologous SLC sequences.

**Table S1. Prediction of the effects of SLC38A8 mutations using the PolyPhen-2 server.** A score > 0.339 is considered detrimental.

| **Substitution** | **Prediction** | **HumDiv Score** | **HumVar Score** |
| --- | --- | --- | --- |
| M34R | Benign | 0.225 | 0.121 |
| E233K | Probably Damaging | 1.000 | 1.000 |
| V236D | Probably Damaging | 1.000 | 0.977 |
| **D283A** | **Probably Damaging** | **1.000** | **1.000** |
| S336A | Benign | 0.001 | 0.000 |
| L344C | Possibly Damaging | 0.962 | 0.652 |
| G412R | Probably Damaging | 1.000 | 1.000 |

**Table S2. Prediction of the effects of SLC38A8 mutations using the SIFT server.**

| **Substitution** | **Prediction** | **Score** | **Median Info** | **Number of sequences at position** |
| --- | --- | --- | --- | --- |
| M34R | Damaging | 0.01 | 3.05 | 17 |
| E233K | Tolerated | 0.35 | 3.06 | 18 |
| V236D | Damaging | 0.03 | 3.06 | 18 |
| **D283A** | **Damaging** | **0.00** | **3.06** | **18** |
| S336A | Tolerated | 0.83 | 3.06 | 18 |
| L344C | Tolerated | 0.20 | 3.17 | 16 |
| G412R | Damaging | 0.00 | 3.10 | 16 |

**Table S3. Prediction of the effects of SLC38A8 mutations using the PROVEAN server.** A score < -2.5 is considered to be deleterious.

| **Substitution** | **Prediction** | **PROVEAN Score** |
| --- | --- | --- |
| M34R | Deleterious | -3.611 |
| E233K | Neutral | -2.267 |
| V236D | Deleterious | -6.274 |
| **D283A** | **Deleterious** | **-7.245** |
| S336A | Neutral | -0.001 |
| L344C | Neutral | -0.440 |
| G412R | Deleterious | -5.317 |

**Supplemental Methods**

*Primary sequence analysis* – Our patient mutation was detected by whole exome sequencing, performed by the New York Genome Center. Exome capture was performed using the Agilent SureSelect XT Human All Exon V_5_+UTRs target enrichment kit. The captured exome was then sequenced using an Illumina HiSeq 2500 with 125 basepair reads. In order to predict whether our patient mutation, as well as other FHONDA mutations, had potential deleterious effects on SLC38A8 structure and function, we submitted the SLC38A8 sequence and mutations to the PolyPhen-2 (Adzhubei, et al., 2013), SIFT (Sim, et al., 2012), and PROVEAN (Choi and Chan, 2015) servers. Multiple sequence alignments were performed in Geneious R8 (Kearse, et al., 2012).

*Homology-based modeling of SLC38A8* – We first took a homology-based approach using the crystal structure of bacterial arginine/agmantine antiporter AdiC (PDB ID: 3OB6) from *E. coli* (Kowalczyk, et al., 2011). We chose the AdiC structure as a homology modeling template because it is a functionally-similar channel that has been recently used as a template to model and validate the membrane topological structure of SNAT4, a related SLC channel expressed in the liver (Kowalczyk, et al., 2011; Shi, et al., 2011; Ma, et al., 2012). The sequence of wild-type (WT) SLC38A8 was modeled onto the template structure using the homology modeling protocol in the YASARA 15.7.25 software package (Krieger, et al., 2009). A total of 5 initial models were generated from the template to generate a hybrid model for further refinement. The hybrid model was refined with an energy minimization in the YAMBER3 (Krieger, et al., 2004) force field followed by a steepest descent minimization and simulated annealing. The resultant homology model of WT SLC38A8 superimposed with the template with an RMSD of 6.9 Å over 274 Cα atoms.

*Structural modeling and analysis of SLC38A8 –* A PSI-BLAST of the SLC38A8 sequence against structures in the Protein Data Bank (PDB) did not yield any templates with ≥ 30% homology. The membrane topological structure of SLC38A8 was modeled using TMHMM (Krogh, et al., 2001). Due to the lack of structural templates in the PDB, the tertiary structure of SLC38A8 was modeled using an threading approach with the I-TASSER program (Zhang, 2008; Roy, et al., 2010; Yang, et al., 2015a; Yang and Zhang, 2015). Additional modeling of SLC38A8 structure was performed using a homology-based approach for comparison (see Supplemental Methods; Fig. S1). The output model from I-TASSER had a confidence score of -0.54 and corresponded well to the membrane topological map generated with TMHMM. Mutational analysis using this model was performed with the FoldX program (Schymkowitz, et al., 2005). First, the RepairPDB function in FoldX was used to adjust the position of residues with bad torsion angles or VanderWaal’s clashes. Free energy calculations were then performed after introducing mutations into SLC38A8 (5 runs per mutation). PyMOL generated all structural figures (Schrödinger Corporation, 2014).

*Electrostatic Calculations* – Charges and hydrogens were added to the WT and mutant SLC38A8 models using PDB2PQR (Dolinsky, et al., 2004). Electrostatic potentials were calculated using the linearized Poisson-Boltzmann equation in the APBS program (Baker, et al., 2001). Protein and solvent dielectric constants were set to 2.0 and 78.0, respectively.

**Supplemental References**

1. Adzhubei I, Jordan DM, Sunyaev SR. 2013. Predicting functional effect of human missense mutations using PolyPhen-2. Curr Protoc Hum Genet *Chapter 7*:Unit7 20.
2. Baker NA, Sept D, Joseph S, Holst MJ, McCammon JA. 2001. Electrostatics of nanosystems: application to microtubules and the ribosome. Proc Natl Acad Sci U S A *98*:10037-10041.
3. Choi Y, Chan AP. 2015. PROVEAN web server: a tool to predict the functional effect of amino acid substitutions and indels. Bioinformatics *31*:2745-2747.
4. Dolinsky TJ, Nielsen JE, McCammon JA, Baker NA. 2004. PDB2PQR: an automated pipeline for the setup of Poisson-Boltzmann electrostatics calculations. Nucleic Acids Res *32*:W665-667.
5. Kearse M, Moir R, Wilson A, Stones-Havas S, Cheung M, Sturrock S, Buxton S, Cooper A, Markowitz S, Duran C, Thierer T, Ashton B*, et al.* 2012. Geneious Basic: an integrated and extendable desktop software platform for the organization and analysis of sequence data. Bioinformatics *28*:1647-1649.
6. Kowalczyk L, Ratera M, Paladino A, Bartoccioni P, Errasti-Murugarren E, Valencia E, Portella G, Bial S, Zorzano A, Fita I, Orozco M, Carpena X*, et al.* 2011. Molecular basis of substrate-induced permeation by an amino acid antiporter. Proc Natl Acad Sci U S A *108*:3935-3940.
7. Krieger E, Darden T, Nabuurs SB, Finkelstein A, Vriend G. 2004. Making optimal use of empirical energy functions: force-field parameterization in crystal space. Proteins *57*:678-683.
8. Krieger E, Joo K, Lee J, Lee J, Raman S, Thompson J, Tyka M, Baker D, Karplus K. 2009. Improving physical realism, stereochemistry, and side-chain accuracy in homology modeling: Four approaches that performed well in CASP8. Proteins *77 Suppl 9*:114-122.
9. Krogh A, Larsson B, von Heijne G, Sonnhammer EL. 2001. Predicting transmembrane protein topology with a hidden Markov model: application to complete genomes. J Mol Biol *305*:567-580.
10. Ma D, Lu P, Yan C, Fan C, Yin P, Wang J, Shi Y. 2012. Structure and mechanism of a glutamate-GABA antiporter. Nature *483*:632-636.
11. Roy A, Kucukural A, Zhang Y. 2010. I-TASSER: a unified platform for automated protein structure and function prediction. Nat Protoc *5*:725-738.
12. Schrödinger Corporation. 2014. *PyMOL* [Online]. New York. Available: http://www.pymol.org/ [Accessed Jul 1 2016].
13. Schymkowitz J, Borg J, Stricher F, Nys R, Rousseau F, Serrano L. 2005. The FoldX web server: an online force field. Nucleic Acids Res *33*:W382-388.
14. Shi Q, Padmanabhan R, Villegas CJ, Gu S, Jiang JX. 2011. Membrane topological structure of neutral system N/A amino acid transporter 4 (SNAT4) protein. J Biol Chem *286*:38086-38094.
15. Sim NL, Kumar P, Hu J, Henikoff S, Schneider G, Ng PC. 2012. SIFT web server: predicting effects of amino acid substitutions on proteins. Nucleic Acids Res *40*:W452-457.
16. Yang J, Yan R, Roy A, Xu D, Poisson J, Zhang Y. 2015a. The I-TASSER Suite: protein structure and function prediction. Nat Methods *12*:7-8.
17. Yang J, Zhang Y. 2015. I-TASSER server: new development for protein structure and function predictions. Nucleic Acids Res *43(W1)*:W174-181.
18. Zhang Y. 2008. I-TASSER server for protein 3D structure prediction. BMC Bioinformatics *9*:40.
